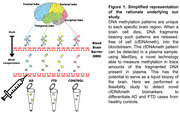# Cell‐Free DNA Methylation: Emerging Non‐Invasive Biomarkers for Liquid Biopsies in Neurodegenerative Disorders

**DOI:** 10.1002/alz70861_108642

**Published:** 2025-12-23

**Authors:** Carolina Ochoa‐Rosales, Stavros Makrodimitris, Carlos Andrés Villegas Lanau, Agustin Ibanez, Claudia Duran‐Aniotz, Duvan Cardona‐Madrigal, David Aguillon, Hieab H.H. Adams

**Affiliations:** ^1^ Latin American Institute for Brain Health (BrainLat), Universidad Adolfo Ibañez, Santiago, Metropolitan Region Chile; ^2^ Erasmus MC, Rotterdam, Holland Netherlands; ^3^ Grupo de Neurociencias de Antioquia, Medellin, Antioquia Colombia; ^4^ Latin American Brain Health Institute (BrainLat), Universidad Adolfo Ibañez, Santiago Chile; ^5^ Latin American Brain Health Institute (BrainLat), Universidad Adolfo Ibáñez, Santiago, Región Metropolitana Chile; ^6^ Latin American Institute for Brain Health (BrainLat), Universidad Adolfo Ibañez, Santiago Chile; ^7^ Neuroscience Group of Antioquia, University of Antioquia, Medellin, Antioquia Colombia; ^8^ Grupo de Neurociencias de Antioquia, Facultad de Medicina, Universidad de Antioquia, Medellín Colombia; ^9^ Radboud University Medical Center, Nijmegen Netherlands

## Abstract

**Background:**

Dying brain cells release DNA fragments, which can be found circulating free of the cell (cfDNA) in the bloodstream. Plasma cfDNA carries a unique cell‐type specific DNA methylation (DNAmeth) signature that could provide information about which specific cells are dying, thus serving as a potential liquid biopsy of the brain (Figure 1). We aimed to perform a pilot study to determine the feasibility of, firstly, identifying DNAmeth patterns specific to brain regions typically degenerated in AD and FTD. Secondly, to identify plasma methylation signatures to differentiate AD and FTD patients from controls.

**Method:**

In Aim 1, we investigated DNAmeth patterns in post‐mortem brain samples across 5 regions (hippocampus, cerebellum, and frontotemporal, occipital, and parietal lobe), from 9 donors diagnosed with AD, FTD, and controls (in a 1:1:1 ratio). In Aim 2, we tested the value of cfDNA methylation in plasma to distinguish diagnoses. Here, we obtained plasma from 15 living participants diagnosed with AD, FTD, and controls (1:1:1). For brain and plasma assessments, we used the novel MeDSeq technique, which can detect methylation at CpG sites in trace amounts of DNA. This is especially suitable due to the lower quantity of cfDNA in plasma samples.

**Result:**

Bioinformatic analyses revealed 353 differentially methylated regions (DMRs) hypermethylated in the hippocampus, while 35 were hypermethylated in the frontotemporal lobe (FDR<0.05). Furthermore, the analysis in plasma cfDNA identified specific methylation signatures across study groups. Specifically, we identified one DMR on chromosome 16q24.2 when comparing dementia cases (AD+FTD) versus controls. Variants at this location have been previously associated with small vessel stroke. Furthermore, we compared AD versus FTD cfDNAmeth, and found 5 significant DMRs close to genes enriched for several lipid‐ and fatty acid‐related processes.

**Conclusion:**

These results demonstrate the feasibility of DNAmeth biomarkers, assessed using MedSeq, in identifying unique DNA regions that are differentially methylated in brain regions relevant to dementia. They also suggest the potential value of cfDNAmeth in plasma as a liquid biopsy biomarker to differentiate dementia types, potentially allowing early AD and FTD diagnosis after clinical validations.